# Chemically Driven Printed Textile Sensors Based on Graphene and Carbon Nanotubes

**DOI:** 10.3390/s140916816

**Published:** 2014-09-10

**Authors:** Ewa Skrzetuska, Michał Puchalski, Izabella Krucińska

**Affiliations:** Department of Material and Commodity Sciences and Textile Metrology, Lodz University of Technology, 90-924 Lodz, Poland; E-Mails: michal.puchalski@p.lodz.pl (M.P.); izabella.krucinska@p.lodz.pl (I.K.)

**Keywords:** graphene, carbon nanotubes, screen printing, textronics

## Abstract

The unique properties of graphene, such as the high elasticity, mechanical strength, thermal conductivity, very high electrical conductivity and transparency, make them it an interesting material for stretchable electronic applications. In the work presented herein, the authors used graphene and carbon nanotubes to introduce chemical sensing properties into textile materials by means of a screen printing method. Carbon nanotubes and graphene pellets were dispersed in water and used as a printing paste in the screen printing process. Three printing paste compositions were prepared—0%, 1% and 3% graphene pellet content with a constant 3% carbon nanotube mass content. Commercially available materials were used in this process. As a substrate, a twill woven cotton fabric was utilized. It has been found that the addition of graphene to printing paste that contains carbon nanotubes significantly enhances the electrical conductivity and sensing properties of the final product.

## 1. Introduction

Textronics is an interdisciplinary science that combines the knowledge of textiles, electronics and informatics and faces new challenges in coordinating and designing novel materials, including nanotechnology-based materials [1]. A key area of interest is related to substrate-integrated circuits for sensing applications involving different types of stimuli, including chemical vapors or temperature changes in human work environments [2]. Recent discoveries in nanoscience and nanotechnology have opened the path for the invention and development of new materials in the field of textronics. Graphene and carbon nanotubes, allotropic forms of carbon, are especially interesting nanomaterials for textronic applications.

Graphene is composed of a two dimensional carbon layer with sp^2^ hybridization, combined in hexagonal units. Due to the sp^2^ hybridization and specific electron configuration, graphene possesses unique properties, such as high mechanical strength (200 times higher than steel), high elasticity, very good electrical and thermal conductivity, and high permeability to light, making it transparent to visible light [3–6].

These properties make graphene an extremely interesting option for applications in modern electronics, including printed electronics. One possible future application, due to its elasticity and durability, is in transparent electrodes and conductive layers, an especially vital application in the case of durable screens, touch panels and OLED screens [7,8]. Graphene is at the same time a prospective material for use as the active elements in mechanical, chemical and biochemical sensors [9,10].

The most adequate form for such applications is the graphene nanopellet (GNP), obtained by physical methods, such as mechanical fragmentation of high quality graphite [10]. In the case of mechanical sensors, the effect of changing percolation paths between GNPs is utilized. Chemical and biochemical activity is mainly caused by graphene defects arising during mechanical fragmentation or chemical reduction of graphene oxide, resulting in active carbon bonds. For chemical and biochemical sensors, detection of target molecules is determined by a change in the observed electrical properties.

Graphene-based sensing systems can be obtained by printing techniques. Printing pastes are prepared by introducing GNPs to a formerly prepared polymer mixture [11] or by introducing graphite by ultrasonic disintegration in N-methylpyrrolidone (NMP) [12]. These system can also be achieved by using graphene oxide based inks and their reduction just after the printing process via chemical [13] or physical methods (e.g., by UV radiation) [14].

In the presented work, the authors have prepared printing pastes by doping multiwall carbon nanotubes (MWCNTs) with graphene pellets. The electrical properties of carbon nanotubes are exploited by many research teams, for instance, in inks produced for printing on foil and paper by means of specific printing methods [15–19].

The authors have focused on the production of a dispersion paste based on commercially available graphene and carbon nanotubes. The physicochemical properties of the obtained materials were similar to those of typical printing pastes for commercial application as electronic printed elements on textile substrates [20]. A screen printing method was chosen due to its high popularity in the production of stretchable and wearable electronics, mainly used for printing planar conductive paths on elastic substrates [20–22].

Printing pastes were based on carbon nanotubes because they are a well-known material for printing conductive paths or textronic sensors [20]. It was assumed that graphene doping would enhance sensing properties due to the formation of defects in the graphene pellet layers.

Additionally, the authors proposed a method for the preparation of the printing pastes, obtained by adding cross-linking ingredients to the aqueous dispersion for stronger bonding of the nanoadditives with the textile substrates. The authors chose to apply cross-linking ingredients due to the lack of information concerning the toxicological effects of nanomaterials deposited on textile substrates. Aliphatic urethane acrylate Ebecryl 2002 (Allnex Belgium SA/NV, Brussels, Belgium) and a photoinitiator (Esacure DP250, Lehmann & Voss & Co., Hamburg, Germany) were used to ensure a durable connection between the paste and substrate.

## 2. Materials and Methods

### 2.1. Nanomaterials

An aqueous dispersion of carbon nanotubes, AquaCyl AQ0301 by Nanocyl (Auvelais, Belgium), was used. It contained c.a. 3.0% MWCNT of the Nanocyl^®^ 7000 series, 90% purity, with nanotube dimensions of 9.5 nm in average diameter and 1.5 μm in average length. Aquacyl AQ0301 is characterized by a surface tension of approximately 58 mN/m, 43 P viscosity and pH = 7, measured by a surface tension analyzer Thermo DCA Radian 315 (Thermo Fisher Scientific, West Palm Beach, FL, USA), rheometer DV3T LV (Brookfield Engineering Laboratories Inc., Middleboro, MA, USA) and pH-meter Elmetron CPC 505 (Elmetron Sp.j., Zabrze, Poland), respectively. The above parameters were determined at 25 °C.

Commercially available graphene pellets were in the form of a dry powder and were purchased from the Graphene Supermarket (Graphene Laboratories Inc., Reading, MA, USA). MO-1 graphene pellets were in the form of 5–30 nm multilayer pellets. This is one is the cheapest, but at the same time highest quality, graphene powders commercially available, obtained by fragmentation of kish graphite. This product is applicable for use in chemical and biological sensors as well as for other applications.

### 2.2. Nanomaterial Characterization

The shape and dimensions of the nanomaterials were investigated by X-ray diffraction, allowing for the collection of average values from a large sample series. Investigation of the carbon nanotubes was conducted by means of small angle X-ray scattering (SAXS). SAXSess equipment equipped with a CuKα source emitting X-rays of wavelength λ = 0.154 nm under a voltage of U_P_ = 50 kV and a current of I_A_ = 40 mA (Anton Paar GmbH, Graz, Austria) was used. Scattering patterns over a 2θ angle range from 0° to 5° were acquired using a CCD camera.

The thickness of and d-spacing between graphene layers of the pellets were determined by wide angle X-ray diffraction (WAXS). An X'PERT PRO from PANanalytical (Panalytica Sp. z o.o., Warszawa, Poland) was used, equipped with a CuKα source emitting X-rays of wavelength λ = 0.154 nm under a voltage of U_P_ = 40 kV and a current of I_A_ = 30 mA. Diffraction patterns covering a 2θ angle range from 0° to 60° were acquired using an X'Celerator counter.

### 2.3. Preparation of the Printing Pastes and the Printing Process

For the preparation of the printing pastes, graphene pellets (MO-1) were introduced at 1 and 3%wt. to an aqueous dispersion of carbon nanotubes (AquaCyl AQ0301).

The composite paste was placed in an ultrasonic tank SONIC 3 (POLSONIC Palczyński Sp.J., Warszawa, Poland) for 15 min, and then, aliphatic urethane acrylate, Ebecryl 2002, were added along with the Esacure DP250 photoinitiator, after which the system was mixed for 0.5 h using a mechanical stirrer RZR1 (Heidolph Instruments GmbH & Co. KG, Schwabach, Germany).

The resulting pastes' surface tension, viscosity and pH were characterized. The results were approximately 65 mN/m, 95 P and pH 7 (at 25 °C), respectively, measured by a surface tension analyzer (Thermo DCA Radian 315), rheometer (Brookfield DV3T LV) and pH-meter (Elmetron CPC 505). No changes in the rheological parameters were observed after adding graphene pellets to the printing paste.

Prepared pastes were applied on a cotton fabric with a twill weave, characterized by a surface mass of 206.3 g/m^3^ and a thickness of 0.410 mm, measured according to the standards EN 29073-1:1992 and EN 29073-2:1992. The apparent density, 503.17 kg/m^3^, was calculated as a ratio of the measured mass per unit area to the fabrics thickness.

The printing process was realized by means of a screen printing machine with an automatic squeegee, MS-300FRO (Printing Machine, Poznań, Poland). Sheets of A4 were printed using a screen with a 49 mesh/cm^2^. The obtained prints were fixed using the cross-linking process by exposing the sample to an IR emitter, type 1384 IR1 250 W Lamp (HELIOS Sp. z o.o., Katowice, Poland) for 30 min.

### 2.4. Surface Resistivity

The electrical properties of the printed woven fabrics were measured according to the standard EN 1149-1:2008 Protective clothing-Electrostatic properties Part 1: Surface resistivity (Test method and requirements). Sample conditioning (24 h) and measurements were performed under isothermal conditions (T = 23 °C) at a relative humidity value of 25% in a conditioning chamber. The electrical resistances of the studied samples were measured along the longitudinal direction (to determine the surface resistance) using a ring electrode (d = 6.9 cm, D = 8.9 cm), a Keithley 610 C electrometer (Keithley Instruments Inc., Cleveland, OH, USA) and a Statron stabilized power supply of unit type 4218 (Statron AG, Mägenwil, Switzerland). According to European Standards, the surface resistivity (ρ_s_) was calculated by the Equation (1):

(1) $$\rho_{s}=kR$$

where R is the mean value of the measured resistances obtained from 5 samples, and k is a geometrical factor based on the electrode equal to 19.8.

### 2.5. Sensor Properties

To test vapor sensitivity, two solvents were selected (acetone and methanol according to standard EN 14605+A1:2010), and measurements were carried out with the use of a laboratory measurement system [23,24]. The sensitivity of each printed fabric to the chosen vapors was evaluated by observing changes in the electrical resistance caused by chemical stimuli. Changes were registered by a special system including a Keithley digital multimeter and the system used for chemical vapor detection, built in The Department of Material and Commodity Sciences and Textile Metrology [23]. The system allows for the determination of the humidity and temperature in the chamber and for the introduction of liquid vapors of a defined concentration. The amount of liquid to be vaporized in the chamber to obtain a vapor of a defined concentration is given by the following Equation (2) [23]:

(2) $$Y=\frac{X\times M}{24.45};M_{c}=Y\times V$$

where *Y* is the liquid density (mg/m^3^), *X* is the ppm content, *M* is the molar mass, *V* is the chamber volume (m^3^), and *M_c_* is the mass of liquid evaporated. The value determined by Equation (2) is stated for the molar volume of a gas under 1 atm pressure at 25 °C [24,25]. After the liquid is evaporated in the gas chamber, the vapor is transmitted to the measuring chamber using a pump, which contains the testing fabric clamped onto the measuring electrodes. Samples for vapor sensitivity measurements had a size of 2 × 4 cm and were attached to electrodes with a size of 4 × 0.5 cm. The pressure used to hold the sample against the electrodes was always the same and was set at a 10 g load. The system allows for vapor concentration changes without unsealing the chamber and can be flushed with nitrogen after each vapor injection. For measurement of the sensing properties, investigations at three concentrations of acetone and methanol were taken—200 ppm, 300 ppm and 400 ppm. The electrical resistance was measured before and after vapor injections. The measuring system used for investigation of vapor textile sensors was described precisely in papers [24,25].

## 3. Results and Discussion

### 3.1. SAXS and WAXS Characterization of the Nanomaterials

**Figure 1.**
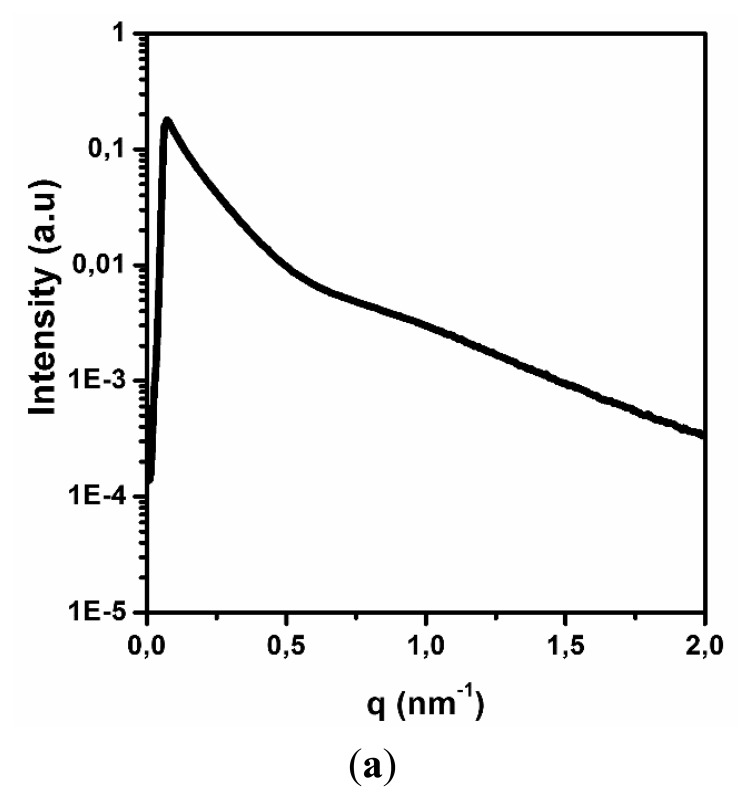

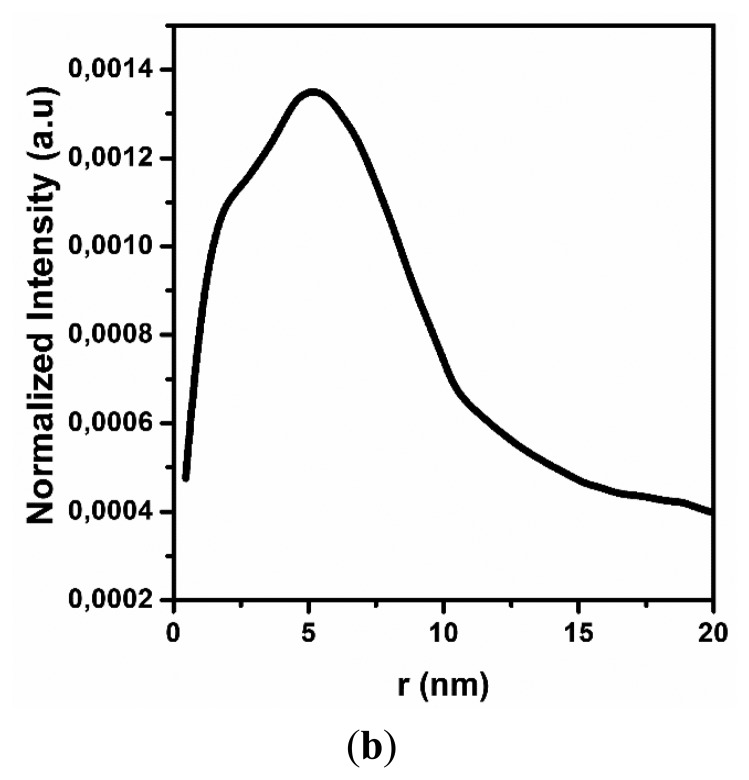
SAXS spectrum of the (**a**) carbon nanotubes and (**b**) their numerical analysis, used to determine the average carbon nanotube diameter.

X-ray techniques are universal and allow for fast analysis of the target sample quality, shape and dimensions. The SAXS spectrum of the carbon nanotubes is shown in Figure 1a. The change in the relative intensity of the spectrum was presented as a function of the wave number q and depends on the 2θ angle according to the following Equation (3):

(3) $$q=\frac{4\pi }{\lambda }\sin\;\left(\frac{2\theta }{2}\right)$$

The obtained SAXS spectrum has a typical shape for cylindrical objects, having a diameter less than 100 nm, similar to MWCNT [26]. This result confirms the quality of the carbon nanotubes and their stability after dispersion. The next step was to evaluate the average diameter of MWCNT in an aqueous solution. Figure 1b contains the results of the numerical analysis from the SAXS spectrum, which used a Fourier transform and electron density measurement method with the GIFT software package. According to the results, the average diameter of the carbon nanotubes is approximately 10 nm, confirming specifications from the manufacturer (Nanocyl).

**Figure 2.**
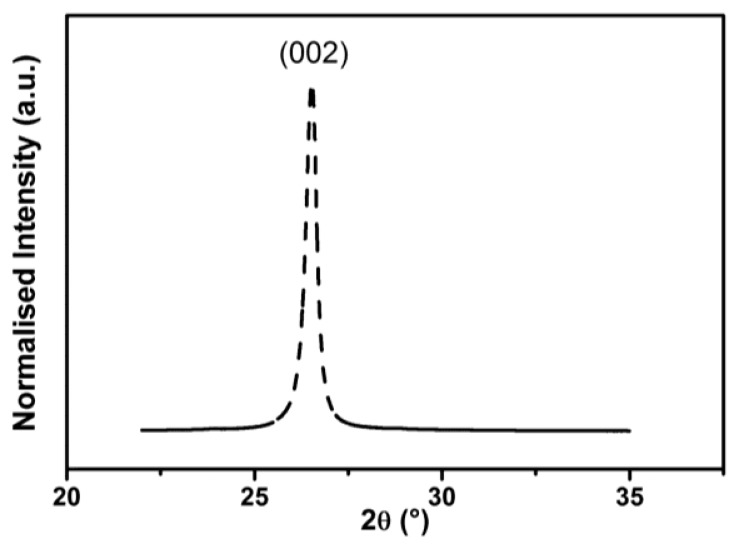
WAXS spectrum for the MO-1 graphene pellets from Graphene Supermarket.

Graphene pellets were characterized by WAXS. In Figure 2, the diffraction pattern is presented. The characteristic diffraction peaks corresponding to the (002) planes of graphite suggest that the thickness of the graphene pellets used was greater than a few monolayers. The obtained X-ray pattern was used to estimate the distance between graphene layers. The d-spacing between two graphene layers was calculated according to Bragg's law (4):

(4) $$z=d=\frac{\lambda }{2\sin\theta }$$

where *d* is the d-spacing in the *Z* direction, *λ* is the X-ray wavelength, and *θ* is the X-ray diffraction angle of the given crystal.

The next step was to determine the thickness of the graphene layer, according to the Scherrer Equation (5):

(5) $${\text{L}}_{\left(\text{hkl}\right)}=\frac{\text{K}\lambda }{\text{B}\cos\theta }$$

where K is the Scherrer constant, 0.9, λ is the X-ray wavelength, θ is the X-ray diffraction angle of the investigated crystal, and B is the full width at half-maximum (FWHM).

The WAXS spectrum analysis allowed for characterization of the commercial graphene pellets. The average measured thickness is 23 nm (68 graphene layers), confirming specifications from the manufacturer (Graphene Supermarket). This is different from standard graphene, which is composed of up to 20 layers to preserve its unique properties. At the same time, the used graphene pellets are commercially available and relatively cheap, which is extremely important from the point of view of commercial applications. The d-spacing between two graphene layers in the GNP materials was estimated. The obtained value of 0.337 nm suggests that this is not a typical graphene (0.335 nm) [27] structure, while the enlarged distance between layers may result in structural defects that will increase the sensitivity to chemical stimuli.

### 3.2. Research on Electrical Resistivity and Sensor Properties

Table 1 shows the mean values of the surface electrical resistance for the printed samples with various graphene content measured at 5 different points on the sample.

The commercially available AquaCyl modified by a cross-linking compound resulted in worse electrical properties than the graphene containing printed samples. At the same time, the increase in graphene content decreases the surface electrical resistivity. It is worth noting that the decrease in surface resistivity is a nonlinear function of GNP addition. The addition of 3% graphene affects the results in the form of a three-fold decrease in resistivity, while the addition of 1% only results in a 30% reduction in resistivity. The presented results confirm that the graphene pellets act as an active modifier of the printing paste based on MWCNT.

Additionally, during the experiment, the amount of graphene was not observed to affect the homogeneity of the final product. The coefficient of variation for the surface resistivity is below 3%, and organoleptic tests indicate no significant differences between studied samples. The influence of the cross-linking compound used, such as aliphatic urethane acrylate and the photoinitiator, was also insignificant, matching results reported in previous work [24,25].

**Figure 3.**
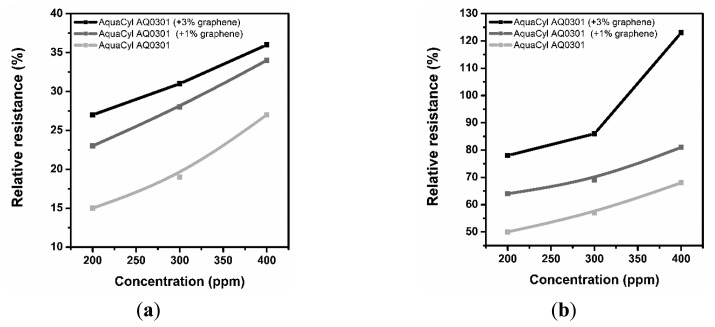
The results for vapor sensing properties of graphene and CNT-based screen printed textiles.

**Figure 4.**
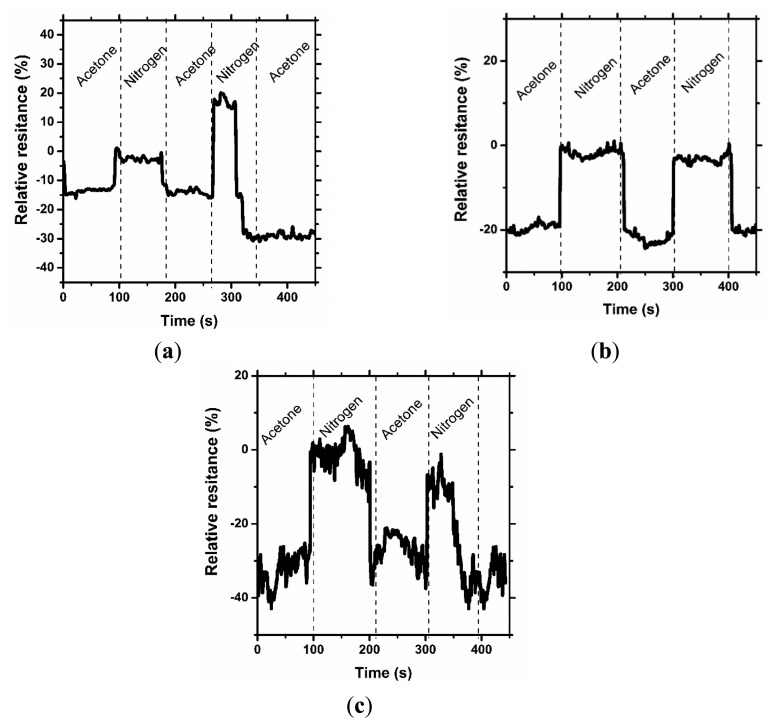
Characteristic sensing properties at 200 ppm acetone vapor for (**a**) AquaCyl AQ0301 + cross-linking compound, (**b**) AquaCyl AQ0301 + 1% graphene + cross-linking compound and (**c**) AquaCyl AQ0301 + 3% graphene + cross-linking compound.

**Figure 5.**
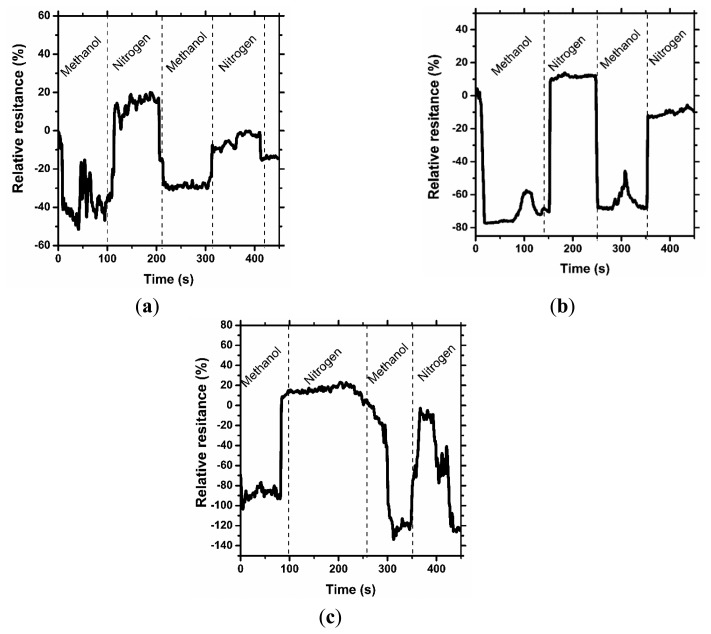
Characteristic sensing properties at 400 ppm methanol vapor for (**a**) AquaCyl AQ0301 + cross-linking compound, (**b**) AquaCyl AQ0301 + 1% graphene + cross-linking compound and (**c**) AquaCyl AQ0301 + 3% graphene + cross-linking compound.

Figures 3, 4 and 5 consolidate the results of the functional investigation of the screen printed textiles. To evaluate sensor properties quantitatively, a relative change in resistance (R_rel_) was used to express the relative change of the screen printed textile's surface electrical conductivity after exposure to chemical stimuli according to Equation (6):

(6) $$R_{\text{rel}}=\frac{R-R_{o}}{R_{o}}$$

where *R_rel_* is the relative resistance, *R_0_* is the initial resistance, and *R* is the final resistance.

Taking into consideration the results from Figure 3, it can be noted that even the addition of 1% graphene has a positive influence on the vapor sensing capabilities. Moreover, the concentration of vapor significantly changes the vapor sensitivity of the screen printed textiles.

Considering the above results, it can be concluded that a change in sensor properties (measured as relative resistance) is proportional to the vapor concentration. Moreover, the studied samples were significantly more sensitive to methanol than to acetone vapors, where the relative change in resistance was above 50% (15% for acetone), and for the samples with a graphene concentration of 3%, it was above 80%. The different intensities of the reaction to various solvent vapors could be correlated to the difference in the electrical dipole moments of the molecules. The dipole moment of methanol is equal to 1.61 D, lower than acetone—2.91 D. Based on the observed results, the obtained printed sensors are more sensitive to solvents with a lower electrical dipole moment, less than 3 D. Hence, it is possible to use screen printed textiles for determining the concentration of chemical vapors.

In Figures 4 and 5, the results of the cyclic sensor sensitivity to acetone and methanol vapors, respectively, are presented. After every vapor injection, lasting approximately 100 s, each sample was rinsed with nitrogen vapor to restore the electrical conductivity to the baseline state. Tests were conducted periodically for each sample, confirming that the sensor properties for chemical stimuli are preserved over time. It was observed that some relaxation time is required for the sensor after exposure to the chemical stimuli to restore its default properties; however, this time is short period, approximately 10 s. More importantly, a decrease in sensor sensitivity after the first cycle is observed. In our opinion, the observed phenomenon is a result of the adsorption of solvent molecules by the printed sensors, which causes a reduction in sensor sensitivity. This phenomenon is the hysteresis for the sensor process and provides important information for future applications and experiments. The results presented in Figure 4a are different from the presented interpretation, and the observations are difficult to interpret. The maximum sensitivity of the prepared sensor without graphene addition to acetone vapor is approximately 20%. This value is close to the critical value of the instruments' experimental sensitivity, and the resulting measurement contains errors.

## 4. Conclusions

The purpose of this article was to report on screen printing pastes for use on textiles substrates. The modification of printing pastes by the addition of carbon allotropes to give them interesting functional properties for specific applications was achieved. These modified pastes can be easily used for textile sensors in chemical manufacturing plants where the risk of vapor poisoning is high, as standalone sensors or as a part of protective clothing. The experimental results on sensor properties with respect to the detection of chemical stimuli proved that these fabricated sensors could be used periodically, after a particular relaxation time, differentiating them from disposable sensors. Possible future research should concentrate on creating a reliable data acquisition system, signal amplification system and analog to digital conversion system that would allow for integration with IT platforms to monitor any risks to humans in the work environment.

## Figures and Tables

**Table 1.** The results for the surface electrical resistivity measurements of the printed samples.

**Ink Content**	**Surface Electrical Resistivity, kΩ (RH = 25%, T = 23 °C)**	**Coefficient of Variation, %**
AquaCyl AQ3001 + cross-linking compound	13.0	2.9
AquaCyl AQ3001 + 0.5% GNPs + cross-linking compound	12.8	2.5
AquaCyl AQ3001 + 1% GNPs + cross-linking compound	9.1	2.2
AquaCyl AQ3001 + 3% GNPs + cross-linking compound	4.7	2.7
